# Acute Pericarditis—Causes—Report of Cases

**Published:** 1876-07

**Authors:** D. A. K. Steele

**Affiliations:** Chicago


### ACUTE PERICARDITIS—CAUSES—REPORT OF CASES.

By D. A. K. STEELE, M.D., Chicago.

(Read before the Chicago Society of Physicians and Surgeons, Feb. 28th, 1876.)

Mr. President, and Gentlemen of the Society :

Presuming that you are thoroughly conversant with the anatomy of the "fountain of life," we will at once proceed to consider some of the causes of an acute pericarditis.

Most frequently this affection is secondary in its development, arising in connection with an attack of rheumatic fever, or in the course of Bright's disease, or resulting from the extension of a contiguous inflammation, as pleurisy or pneumonia; these are certainly the most frequent causes, according to my observation. Among other causes mentioned are low forms of fever, pyæmia, puerperal fever, scarlatina, scurvy, adjacent abscesses, etc. Among primary causes are mechanical violence, penetrating wounds, and "cold," exciting a local inflammation.

CASE I. Pericarditis and Bright's disease—tetanic spasms—death—post-mortem exhibit.

John Crane, miller, aet. 27, German, was admitted to Cook County Hospital Dec. 30th, 1873, during the service of Prof. J. P. Ross. States that he enjoyed good health until seven years ago, when he contracted syphilis, followed by constitutional symptoms. About two and a half years ago, while in the South, had intermittent fever and diarrhœa, for some six months, when he went to New York and was an inmate of a hospital for nineteen months, suffering from general dropsy. Left the hospital four months ago nearly recovered from the dropsy; the right leg still remains a little swollen and has a few ulcers upon it, for the relief of which he was admitted to the ward. After being in the hospital about two weeks, during which time he was up and around the ward, he was attacked with slight rigors. Diarrhœa, nausea and vomiting followed in a few days, with shortness of breath and pain in the region of the heart.

Jan. 17th. On examination find skin hot, tongue dry and brown in centre; appetite poor; patient restless, sleeps poorly. Pulse 120, weak; resp. 20. Auscultation reveals a to-and-fro friction sound all over the præcordial region. Ordered a hot poultice, and gave morphia to control pain. Also quiniæ sulph. gr. iij ter die, and turpentine and laudanum emulsion, with one-thirty-second of a grain of strychniæ sulph., every four hours.

Jan. 18th. Had considerable pain in chest last night, although during the night he took one gr. morphia ; was restless and uneasy ; friction sound still heard ; area of præcordial dullness is increased.

2 P. M. Patient began to be very restless and uneasy ; has slight delirium.

4 P. M. Lost all consciousness and began having tetanic spasms ; head thrown back ; twitching of the muscles of the face and arms ; pulse full and strong ; breathing slow and stertorous ; pupils contracted, but respond to light. Ordered blisters behind the ears, and bromide potash gr. xv every hour.

11 P. M. Patient has had several spasms, his head is thrown back ; still unconscious. On attempting to have him swallow, spasms are induced. Pulse 140, full and strong. Is winking constantly and rolling his eyes ; both arms jerk and twitch ; legs drawn up.

12 M. Had a severe convulsion, and died.

Post mortem 36 hours after death. Pericardium contained about 4 oz. of serum ; abundant recent lymph covering heart and pericardium. Heart hypertrophied ; lungs and brain healthy ; kidneys small and fatty.

CASE II. Idiopathic pericarditis, with effusion—pneumatic aspiration—death—autopsy. (This case was reported in full before the Society, Oct. 12th, 1874.) The following is a synopsis :

Edward S., aet. 45, laborer, Germany ; weight one hundred and seventy pounds ; height five feet eleven inches. Was admitted to the County Hospital Sept. 22nd, 1874, stating that he had been well until ten weeks prior to admission, when, becoming overheated, he took a violent cold. Had several slight chills, followed by high fever and cramping pains in left side in region of the heart ; pain was aggravated by a deep inspiration. Had no cough, appetite poor, bowels costive, breathing labored. This difficulty continued for about four weeks, when he began to convalesce and went to work. In about three weeks, after another exposure to cold, he was again attacked with pain in left chest, similar in character to the first; began to cough, raising a scanty frothy sputum; breath has gradually become shorter for the past two weeks. Is very restless and uneasy. Has never had rheumatism or syphilis.

On admission, find patient a large, well nourished man; lies on left side, with shoulders elevated and thighs flexed on abdomen; breathing hurried and laborious; face anxious and cyanosed; skin cool and moist; tongue flabby, tremulous, and coated brown. Pulse 132, small, thready and irregular. Resp. 28.

Physical examination. Inspection reveals bulging of left chest and præcordial region, partial obliteration of the intercostal spaces and decided loss of motion in left chest; epigastric protrusion; heart communicates no thoracic shock.

Percussion gives complete dullness over a somewhat triangular space in left chest, the base of which would correspond to a transverse line drawn from left axillary region three inches below left nipple to a point two inches to right of xiphoid appendix, apex five inches above and one and a half inches within left nipple. The dullness is but slightly altered by postural change. Behind, fair resonance.

On auscultation, bronchial breathing at apex of left lung; below third rib, an absence of all lung sounds; behind, over apex of left lung bronchial breathing, over middle, broncho-vesicular with mucous rales; below bronchial again, with fine crepitant rales and occasional friction. On listening, over cardiac region find an absence of all heart sounds; along the right border of sternum, a few to-and-fro pericardial friction sounds. Applied warm jacket poultice and gave alcoholic stimuli. Dyspnœa increasing, next morning Prof. H. A. Johnson was summoned, and tapped pericardial sac with the aspirator, through the fifth intercostal space, two inches to the left of sternum, withdrawing about one ounce of bloody serum, with no inconvenience to the patient. He was then given carb. ammonia, quinia and camphor, and the chest painted with tr. iodine.

24th. Expresses himself as feeling a little better; breathes easier. Continued treatment, with but little change of symptoms or physical signs, until 28th, when a pleuro-pneumonia of the left chest manifested itself, with a corresponding increase in the gravity of the symptoms. Warm poultices were kept continuously applied to the chest, with artificial heat to the extremities, and the exhibition of stimulants.

30th. A consultation of the staff was held, and it was determined to again use the aspirator. Dr. Johnson introduced the canula near the point of previous puncture, but carried it a little higher, and on turning the stopcock was rewarded by seeing a full stream of bloody serum flowing from the pericardial sac into the receiving bottle. Eleven and one-half ounces were withdrawn, when the canula was removed. No unpleasant symptoms occurred. The immediate effect of the operation was to relieve the turgescence of the superficial vessels, and steady and strengthen the heart's action. Breathing became easier, and the apex beat of the heart could be seen for the first time, striking a little to the left and higher than normal; cardiac sounds distinctly heard; bulging of intercostal spaces less marked. Half an hour after the operation, pulse 132; resp. 28.

Oct. 1st. Pneumonic inflammation more extensive; extremities cold, radial pulse imperceptible; heart's action rapid, feeble and irregular; general cyanosis; constant nausea and vomiting; gradually sank, and died at one o'clock the following morning.

Autopsy fourteen hours after death. On lifting up sternum, found pericardium immensely distended with fluid, occupying nearly the whole of the left chest, and overlapping the right lung for two or three inches. Diaphragm depressed, moderate pleuritic effusion in both chests. On opening pericardium, found it much thickened and congested, containing a very large amount of bloody serum. Point of last puncture readily seen; point of previous puncture closed by adhesive inflammation. Heart hypertrophied, dilated, and apparently inflamed, outer surface covered with tenacious, coagulated lymph, arranged in segregated layers resembling villi, varying in depth from one-half to one inch, and adhering so firmly to heart as to be with difficulty removed. Pericardium adherent to heart around base by means of this plastic deposit. Left lung throughout pneumonic, in a state of red hepatization, and firmly adherent to chest walls by recent pleuritic adhesions ; right lung congested; kidneys, liver and spleen very much congested; other organs not examined. The heart and sac together weighed four pounds.

CASE III. Rheumatic pericarditis and organic disease of heart—death—post-mortem.

Jan. 4, 1876. I was called to see Jennie S., æt. 11, American, who gave the following family history : Father dead one year, from an overdose of tartar emetic, prescribed by a drunken nurse. Father had been in failing health for a number of years ; was troubled with slight cough, shortness of breath and palpitation of the heart ; was supposed to have heart disease. Mother living and well, although never robust. Four sisters living, all in poor health, three of them suffering from organic disease of the heart, hypertrophy with mitral insufficiency. All of the children had scarlatina when small ; none of them ever had rheumatism, except the little patient, who four years ago, for the first time, suffered from acute articular rheumatism; was treated by Dr. W. T. Montgomery, with alkalies internally, and tinct. iodine and hot fomentations locally; was sick about four months. Every winter since then has had attacks, lasting from a few days to a number of weeks. For past three years has been troubled with palpitation, shortness of breath on slight exercise, and occasional cough. Was treated by me ten months ago for rheumatic endocarditis and pneumonia ; was sick then about three weeks ; ever since has had a loud mitral regurgitant murmur and gradual enlargement of the heart. General health, aside from this, has been good ; has gone to school most of the time. For the past few days has complained of pain in the ankles, knees, wrists and shoulders ; at times feels a little feverish, sleeps poorly. Find joints swoolen and tender, skin hot, tongue furred, pulse somewhat accelerated. Prescribed potassæ acetas, potassic iodide, and tr. digitalis, with Dover's powders at night ; affected joints to be rubbed with chloroform liniment, and swathed in cotton wool.

Jan. 5th. Rested well ; feels a little better.

6th. Complains of gastralgia and nausea. Discontinued the acetate and iodide of potash, and substituted the bicarb. of soda. Ordered morph. sulph., gr. $\frac{1}{8}$, as required to relieve pain.

7th. Gastric irritability less, although still no desire for food. Pulse, 110, full. Joints not so painful ; has a troublesome dry cough ; lung sounds about normal, left lung a little feeble.

8th, A. M. Very restless last night ; complains of pain in region of heart this morning. On making a stethoscopic examination of chest, find a harsh to-and-fro pericardial friction sound over nearly the whole præcordial region ; valvular murmur more distinct ; heart's action strong and full. Applied hot flaxseed poultice to the entire left chest. P. M. Exquisite pain over the entire præcordia ; breathing hurried ; eyes congested ; tongue heavily furred ; pulse, 130. Gave saline cathartic, and contiued hot flaxseed meal poultices.

9th. Dr. S. A. McWilliams saw the patient with me. Pain is excruciating ; constant dry cough. Requires hypodermic injections of morphia, gr. $\frac{1}{8}$, morning and evening, to procure any rest. Ordered, pot. iodide, gr. v ; tr. digitalis, gtts. xv ; tr. gelseminum, gtts. vij ; syr. prunus. virg., ʒj, every three hours. Applied fly blisters locally.

15th. For the past week the above treatment has been continued, with the occasional substitution of potassic bromide for the morphia, which, however, does not control the pain. The general condition of the patient remains about the same. Pulse a little above 100 and rather feeble. Physical signs unchanged.

16th. No pain in joints; severe pain through the temples and in præcordia. She takes milk, beef tea and wine freely. Bowels have been kept soluble with citrate of magnesia.

20th. Physical signs as before, except that the friction sound is not so distinct; heart's action more feeble, and occasionally intermits; cerebral pain very severe. At times is in a state of maniacal excitement, occasional delirium; rests only for a short time, and then under the influence of morphia; takes nourishment pretty well.

21st. Passed a very restless night; constantly sits up with the chest inclined forward; sleeps in this position; can not lie down; says she can not breathe if she does, face becomes purplish and anxious when she attempts it. Pulse, 115, intermittent and feeble. Get increased cardiac dullness in front; friction sound not so distinct; heart's impulse still seen and felt. Applied emplast cantharidis to chest.

22d. Symptoms unchanged. Chest so tender as not to admit of an examination; covered it with cotton wool and oil silk.

23d. Pulse, 120, small, weak and irregular; respiration labored; facial expression more anxious. On examination, decided loss of motion in left chest; grazing pericardial friction still present, but feeble as compared with that first heard. P. M. Delirious; gradually sank, and died at nine o'clock.

The mother, an intelligent lady, readily granted permission to hold a post-mortem examination, and twenty hours after death I made the autopsy, in the presence of Dr. P. S. Hayes and Messrs. Beale, Bean and Warren, students of medicine. Incision from manubrium to umbilicus, down mesial line. Both pleural cavities contained a considerable quantity of serum. Although there were no evidences of a recent inflammation, a few old pleuritic adhesions were found. Right lung normal; left lung small and somewhat atrophic; crepitates throughout; no tubercle. Pericardium firmly adherent to the heart in front and at the sides; behind distended, with flakey serum to its utmost extent. Behind it was intensely congested, and at one point seemed almost gangrenous. On laying open the sac, found the firm adhesions from recent bands of lymph already noted. (These adhesions readily explain why we got the cardiac sounds and frictions so distinctly after the effusion occurred. Ordinarily, after a pericardial effusion, the heart sounds become feeble or are lost, and the impulse disappears.) Heart and pericardium weighed 20½ ounces. Weight in this patient should not have exceeded nine or ten ounces. On opening the left ventricle, find mitral valve thickened and roughened by a deposit about the margins of the curtain, while just within the left auricle there is a patch about the size of a silver half dollar, of rough, thickened deposit, in the endocardium. The endocardium is red and inflamed, while the muscular tissue of heart was unusually vascular, probably the seat of a rheumatic inflammation. Stomach apparently healthy. Liver enlarged and congested. Other organs not examined.

These cases, you will observe, were all connected with some other grave disease.

